# The complete mitochondrial genome sequence of *Abudefduf Bengalensis* (Perciformes; Pomacentridae)

**DOI:** 10.1080/23802359.2018.1511855

**Published:** 2018-10-08

**Authors:** Wei Shi, Hairong Luo, Hui Yu

**Affiliations:** a College of Life Science, Foshan University, Foshan, Guangdong, China;; b CAS Key Laboratory of Tropical Marine Bio-resources and Ecology, South China Sea Institute of Oceanology, Chinese Academy of Sciences, 164 West Xingang Road, Guangzhou, China

**Keywords:** Perciformes, *Abudefduf bengalensis*, phylogenetic relationship

## Abstract

The complete mitochondrial genome of marine fish *Abudefduf bengalensis* was sequenced by the high throughput sequencing method. The genome of this species is 16,776 bp in length, consisting of 13 protein-coding genes, 22 tRNA genes, two rRNA genes and one large non-coding region. The gene arrangement of *A*. *bengalensis* is identical to that of other common fishes. Phylogenetic tree based on 13 protein-coding genes shows that the clade of genus *Abudefduf* has a closer phylogenetic relationship to the lineage of genus *Amphiprion* and *Premnas biaculeatus* than to *Labracinus cyclophthalmus*.

*Abudefduf bengalensis* (Bloch, 1787), common name Sergeant-major or Bengal sergeant, is a kind of reef-associated fishes (Allen 1991). It has a whitish to pale greyish sergeant major with six or seven narrow black bands on the body, usually inhabits in brackish or coastal waters of Indo-West Pacific Ocean, from Pakistan and India to Andaman Sea, Papua New Guinea, Australia to Japan (Masuda et al. 1984). Adults occur singly or in small groups in inshore reef and lagoon environments, with highly territorial. They feed on algae, gastropods, and small crabs (Lieske and Myers 1994).

This study first reported the complete mitochondrial genome of *A*. *bengalensis*, and analyzed its phylogenetic relationship with some other species from other families of Perciformes, based on samples collected from Naozhou Island in Zhanjiang, China (geographic coordinate: N 20°88′, E 110°56′). The whole body specimen was preserved in ethanol and registered to the Marine Biodiversity Collection of South China Sea, Chinese Academy of Sciences, under the voucher number SW20181071706.

The complete mitochondrial genome of *A*. *bengalensis* is 16,776 bp in length (GenBank accession No. MH678614), including 13 protein-coding genes, two rRNA genes, 22 tRNA genes, one OL (origin of replication on the light-strand) and one D-Loop (control region). The OL is 47 bp in length, located in the cluster of five tRNA genes (WANCY region) between tRNA-Asn and tRNA-Cys. The D-loop is 1065 bp in length, located between *tRNA*-*Pro* and *tRNA*-*Phe*. Gene arrangement of this genome is identical to that of common fish, and most genes in this genome are encoded by the heavy strand (H-strand), except for *ND6* and eight tRNA genes (Boore 1999; Yu and Kwak 2015; Gong et al. 2017). Overall base composition values for the mitochondrial genome are 29.4%, 29.1%, 15.7%, and 25.8% for A, C, G, and T, respectively.

The phylogenetic relationships of *A*. *bengalensis* with 13 closely related species were analyzed in this study. Complete mitochondrial genes of these 14 species were available on GenBank. The Maximum Likelihood evolutionary tree (ML tree) was constructed by MEGA 7 (Kumar et al. 2016) based on 1st and 2nd codon sequences of 13 protein-coding genes.

In the ML phylogenetic tree, *A*. *bengalensis* clustered with species of same genus, *Abudefduf vaigiensis*. Ten species of *Amphiprion*, *Amphiprion ocellaris*, *Amphiprion bicinctus*, *Amphiprion polymnus*, *Amphiprion percula*, *Amphiprion clarkii*, *Amphiprion frenatus*, *Amphiprion perideraion*, *Amphiprion ephippium*, *Amphiprion akallopisos* and *Amphiprion sebae*, were all classified into one lineage, as a sister lineage to species of genus *Premnas*, *P*. *biaculeatus*, with a strong support. The clade of genus *Abudefduf* has a closer phylogenetic relationship to the former mentioned lineage of genus *Amphiprion* and *P*. *biaculeatus*, than to *Labracinus cyclophthalmus* (Figure 1). The results of this study show that genus *Amphiprion* has a closer phylogenetic relationship to genus *Premnas* than to genus *Labracinus*.

**Figure 1.**
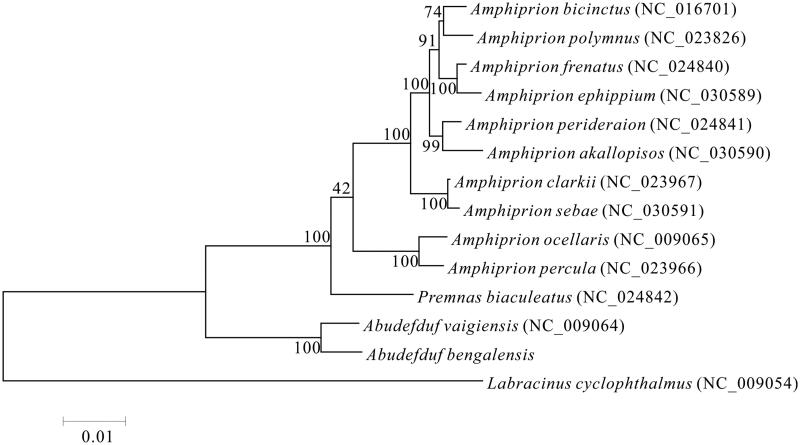
Maximum likelihood phylogenetic tree was constructed based on 1st and 2nd codon sequences of 13 protein-coding genes of 14 species.
